# When the place of dying is lost: Reflections on care, uncertainty, and the limits of intervention

**DOI:** 10.1017/S1478951526102557

**Published:** 2026-04-30

**Authors:** Miguel Augusto-Rodrigues, João Carlos Geber-Júnior

**Affiliations:** 1 Faculty of Medicine of Ribeirão Preto (FMRP), University of São Paulo, São Paulo, Brazil; 2 Center for Oncologic Intercurrence Care (CAIO), Cancer Institute of the State of São Paulo (ICESP), São Paulo, Brazil

I did not learn about end-of-life care from textbooks. I learned it at the bedside of my great-grandfather.

He had been ill for years. His body, once strong from a life rooted in rural labor, gradually became confined to a bed. Over time, clinical action intensified: home visits, hospitalizations, and repeated medical interventions. Everything that could be done was done. Yet, alongside this accumulation of medical response, something quieter persisted – a repeated and simple wish: he wanted to die at home.

He did not.

He died in the hospital, surrounded by interventions aimed at sustaining him, yet distant from the place that had shaped his life and identity. In the final days, he was no longer able to speak for himself, and decisions unfolded around him rather than with him. At the time, this outcome was experienced by the family as inevitable. Only later did it become, for me, a question.

If his wish was known, and if care was continuously present, why was it not possible for him to die where he wanted?

This question does not arise from accusation, but from an unease that remains unresolved: how can care remain present, and yet fail to honor what mattered most?

Medical training emphasizes recognition, diagnosis, and intervention. We are taught to act under uncertainty, to escalate treatment when deterioration occurs, and to avoid omission. In this framework, action is often equated with responsibility. However, we are less frequently taught how to recognize when continued intervention no longer serves the person in front of us, but rather the internal logic of disease progression.

Uncertainty, in this sense, is not a failure of medicine – it is one of its defining conditions. Prognosis is rarely precise, trajectories are heterogeneous, and decisions unfold over time rather than at a single decisive moment. Prognosis, therefore, should not be understood as a discrete act of prediction, but as a longitudinal process of alignment between clinical evolution and the goals of care (Geber-Junior and Forte 2025a). Yet precisely because of this, uncertainty should not justify the indefinite continuation of interventions. Instead, it should call for a more attentive and reflexive form of clinical presence – one capable of revisiting goals of care as the patient’s condition evolves.

The experience of my great-grandfather’s illness illustrates this dilemma. From a technical standpoint, there was no evident failure. He received medical attention, his symptoms were addressed, and decisions were made within the limits of what was known at each moment. However, from a relational and existential perspective, something was lost: the alignment between the care provided and the meaning of that care for the person receiving it.

Cassell (1982) reminds us that suffering cannot be reduced to physical symptoms; rather, it involves a perceived threat to the integrity of the person. To be removed from one’s place of belonging at the end of life may not be captured by laboratory values or clinical scores, yet it may represent a profound dimension of suffering. In parallel, work in palliative care has emphasized that meaning, dignity, and relational presence are not secondary to treatment – they are integral to what defines good care (Breitbart et al. 2010).

What this case made visible to me was not a failure to act, but a difficulty in reorienting action. The momentum of medical care, once initiated, tends to perpetuate itself. Each intervention leads to another; each hospitalization opens the possibility of further treatment. Within this trajectory, the question of “whether to continue” is often replaced by “what to do next.”

Yet good clinical practice may depend precisely on recovering that first question.

To care is not only to intervene, but to discern when intervention no longer corresponds to the needs, values, or possibilities of the patient. This discernment is not a passive act, nor does it represent therapeutic abandonment. On the contrary, it requires an active, ethically grounded engagement with the patient’s evolving condition. It demands the ability to remain present in situations where medicine cannot offer cure or control, but can still offer meaning, accompaniment, and relief (Geber-Junior and Forte 2025b).

In this context, the place of death becomes more than a logistical outcome; it becomes a reflection of whether care remained aligned with the person. Dying at home is not inherently superior to dying in a hospital. However, when a clear and persistent preference is expressed and not achieved, it invites us to examine how decisions were made, how goals were revisited, and how the patient’s voice was integrated into the process.

Importantly, this is not a critique of individual clinicians or isolated decisions. Rather, it reflects a structural feature of modern medicine: its orientation toward intervention, often sustained by the ethical imperative to act. This orientation, while fundamental to many therapeutic successes, may also obscure the moment when action should give way to reorientation.

Looking back, I do not conclude that something wrong was done. Instead, I understand that something essential may not have been recognized in time. The challenge for clinicians is not only to respond to disease, but to remain attentive to the shifting meaning of care itself.

This requires a reframing of clinical responsibility. Responsibility is not exhausted by doing what can be done; it also involves recognizing when what can be done no longer aligns with what should be done. In this sense, the most important transitions in medicine are not always between treatments, but between intentions.

We are trained to extend life, and often we succeed. But there are moments when the task is different: to ensure that the life that remains is lived in accordance with the person’s values, and that its ending is not displaced from the context to which it belongs.

Sometimes, the failure of care lies not in doing too little, but in failing to recognize when doing more no longer means caring better.
